# “Foot and Ankle Risk Factors for Future Falls in Older Adults: A Systematic Review With Meta‐Analysis of Prospective Cohort Studies”

**DOI:** 10.1002/hsr2.73007

**Published:** 2026-08-13

**Authors:** Samuele Valeriani, Gianluca Melotto, Thanaporn Tunprasert

**Affiliations:** ^1^ School of Education, Sport and Health Sciences University of Brighton Brighton UK; ^2^ School of Health & Rehabilitation Science Health Sciences University Bournemouth UK

**Keywords:** fall risk factors, falls, foot and ankle problems, older adults, systematic review

## Abstract

**Background:**

Falls represent a major cause of morbidity and healthcare costs in older adults. Previous systematic reviews have identified certain foot and ankle problems as potential fall risk factors in older adults, but evidence has been predominantly cross‐sectional, limiting causal inference. This review evaluates which foot and ankle problems are prospectively associated with falls in older adults across various settings.

**Methods:**

Six electronic databases were searched from inception to October 2025. Prospective cohort studies on foot and ankle problems and fall risk in adults aged ≥ 60 years were included. Reviewers screened for eligibility, extracted data of included articles and assessed methodological quality using the National Institute of Health (NIH) tool. Meta‐analyses were conducted using random‐effects models when appropriate. Narrative synthesis was used when pooling was not feasible.

**Results:**

A total of 2,189 unique articles were screened, and 14 studies met the eligibility criteria. Meta‐analyses showed significant associations between increased fall risk and foot pain (pooled OR = 1.99; 95% CI: 1.38–2.88), impaired sensory function (pooled OR = 1.52; 95% CI: 1.37–1.68) and intrinsic foot muscle weakness (hallux plantarflexor: pooled OR = 1.65; 95% CI: 1.14–2.38) (lesser toes plantarflexor: pooled OR = 2.04; 95% CI: 1.43–2.92). Findings for deformities, ankle range of motion, lesions, posture and general foot problems were inconsistent or based on limited data. The methodological quality of the included studies was variable, and a common limitation was the lack of adjustment for confounders.

**Conclusions:**

Foot pain, impaired sensory function and intrinsic foot muscle weakness are associated with an increased fall risk in older adults. These findings reinforce the importance of including foot health assessment within fall prevention strategies. Future research should focus on developing and validating screening tools for foot‐related risk factors to help clinicians identify what to assess and guide targeted interventions.

## Summary

- Foot pain, impaired sensory function and intrinsic foot muscle weakness are prospectively associated with an increased risk of future falls in older adults
- Integrating structured foot/ankle assessment (pain, monofilament sensation, toe/hallux strength) into routine fall‐risk evaluations may improve early identification of at‐risk older adults.
- The methodological quality of the available evidence was generally low, due to the lack of adjustment for confounders and the heterogeneity in assessment methods, highlighting the need for more rigorous prospective research.

**Why Does This Paper Matter?:**

- Foot and ankle conditions are common, under‐recognized, and often modifiable contributors to fall risk in older adults. By synthesizing only prospective evidence, this review identifies foot pain, sensory loss and intrinsic muscle weakness as predictors of future falls. These findings support integrating structured foot assessment into routine fall‐risk evaluations and highlight the need for more rigorous studies in this often overlooked area.

## 1 Introduction

Falls among older adults are a global public health problem, as they are a major cause of morbidity and mortality in this population [1, 2, 3, 4]. There is a consensus that falls are a multifactorial phenomenon resulting from a combination of intrinsic and extrinsic factors and that having more risk factors increases the likelihood of falling [5]. Among the over 150 identified risk factors, emerging evidence suggests that foot and ankle problems are included as significant ones [6, 7].

Foot and ankle problems encompass pain, musculoskeletal disorders, deformities, sensory deficits, and dermatological issues, all highly prevalent in older adults [8]. These changes may impair balance, functional ability and gait, thereby increasing fall risk [9]. Current fall prevention guidelines recommend assessing foot problems as part of a multifactorial falls risk assessment [10]. However, the lack of specific recommendations on which aspects to assess and how, reflects a gap in the current literature.

The previous systematic review with meta‐analysis by Menz et al. [7] provided valuable insights regarding the association between foot and ankle problems and fall risk in community‐dwelling older adults. By including both cross‐sectional and prospective studies, it provided a broad overview of the evidence available at that time. However, cross‐sectional studies are limited in inferring causality, as they cannot establish temporal relationships between exposure and outcome [11]. This limitation is particularly relevant in fall risk research, where understanding the sequence of events is crucial [6]. These considerations, together with the growing prospective evidence published in recent years, suggested the need for a new review focusing solely on longitudinal studies. To address this gap, the present systematic review and meta‐analysis aimed to synthesize prospective evidence on foot and ankle problems as risk factors for falls in older adults. Furthermore, to better capture the current state of research in this area, all settings have been included.

## 2 Methods

### 2.1 Review Design

The following systematic review was performed in line with the Preferred Report Items for Systematic Review and Meta‐Analyses (PRISMA) statement [12] and a protocol was structured a priori and registered with PROSPERO in May 2024 (ID CRD42024499402). Deviations from the protocol are reported and justified in Supporting Information: S1.

### 2.2 Search Strategy

A comprehensive search was conducted on June 1, 2024, and updated on October 24, 2025, with no additional eligible studies identified. The following electronic databases were searched: CINAHL, EMBASE (via Ovid), MEDLINE (via Ovid), Web of Science, Scopus, and the Cochrane Library. The search included all studies from database inception to the search date, with no language restrictions. For non‐English articles, the authors were contacted for translations. If translation was not feasible, those studies have been reported as “studies awaiting classification” and included in the PRISMA flow diagram as recommended by the Cochrane Handbook for Systematic Reviews of Interventions [13]. Detailed search strategies for each database are provided in Supporting Information S2.

### 2.3 Study Inclusion and Exclusion Criteria

Inclusion and exclusion criteria were defined a priori using the PECO framework [14]. A comprehensive list of inclusion and exclusion criteria is provided in Table 1.

**Table 1:** Inclusion and exclusion criteria for study screening.

	Inclusion criteria	Exclusion criteria
Population	‐ Adults ≥ 60 years	‐ Adults < 60 years
		‐ Unspecified age range
		‐ Selectively recruited by condition or demographic (e.g., sex, ethnicity, occupation)
Exposure	‐ All foot/ankle problems and assessment methods	‐ Studies not addressing foot/ankle problems
Comparator	‐ With versus without foot/ankle problems	‐ No clear distinction between the groups
	‐ Fallers versus non‐fallers (within foot/ankle problems)	
Outcome	‐ Falls defined as ‘unintentional’ events	‐ Intentional falls
	‐ Fall data: incident count or risk measures (e.g., OR, RR, IRR, HR, RD, SMD)	‐ No definition of ‘fall’ given
		‐ Falls caused by significant external forces (e.g., vehicle impact)
Study Design	‐ Prospective cohort study, with or without control group	‐ Any study design other than prospective cohort
	‐ Follow‐up ≥ 6 months	‐ Follow‐up < 6 months
Setting	‐ Any setting	

Abbreviations: HR, hazard ratio; IRR, incidence rate ratio; OR, odds ratio; RD, risk difference; RR, risk ratio/relative risk; SMD, standardized mean difference.

This review included only studies with participants aged 60 years or older, according to the World Health Organization's (WHO) threshold for classifying ‘older adults’ [15]. Studies without a clearly defined age range were excluded to avoid confounding from younger participants who are more likely to experience falls due to external factors linked to higher activity levels [16]. In addition, studies selectively recruiting based on specific medical conditions or characteristics (e.g., sex, socioeconomic status, or occupation) were excluded to maintain generalizability of the findings. All settings were deemed eligible, including community, residential care and hospital settings.

The exposure of interest included foot and ankle problems of various aetiologies, encompassing, but not limited to, structural deformities, musculoskeletal issues, dermatological conditions, neurological disorders, and pain. Studies that did not specifically address foot and ankle problems were excluded. Comparators included individuals with foot and ankle problems versus those without, and fallers versus non‐fallers within the exposed population. Studies lacking a clear distinction between comparator groups were excluded.

The primary outcome was the risk of falls among older adults, measured by effect sizes such as odds ratios (OR), risk ratios (RR), Incidence Rate Ratios (IRR), Hazard Ratios (HR), or Standardized Mean Differences (SMD). Falls had to be defined as "unintentional events". Only prospective cohort studies with a minimum follow‐up duration of 6 months were considered eligible, as this study design offers higher‐quality observational evidence and improves causal inference by establishing temporal relationships between exposure and outcome [17].

### 2.4 Study Selection Process

Titles and abstracts were screened independently by two reviewers (S.V. and G.M.) based on the eligibility criteria. Discrepancies were resolved through discussion or by involving a third reviewer (T.T.). Full texts of potentially relevant articles underwent a second screening stage. Backward and forward reference searching were conducted to identify additional studies [13].

### 2.5 Data Extraction

Data extraction was conducted independently by two reviewers (S.V. and G.M.). Disagreements were resolved through discussion or by involving a third reviewer (T.T.). Data extracted included study characteristics (e.g., publication year, sample size, setting), participant demographics (e.g., age, sex), and methods used to define and assess falls and foot and ankle problems.

Effect sizes extracted included ORs, RRs, IRRs, HRs, RDs and SMDs, along with their 95% confidence intervals (CI). Both unadjusted and adjusted estimates were recorded, along with the variables used for adjustment.

Foot and ankle problems were grouped into eight categories, adapted from Menz et al. [7]: foot/ankle pain, sensory function, muscle strength, deformity, lesions, range of motion (ROM), posture and general problems.

Authors were contacted for missing or unclear data. When possible, raw data were used to calculate effect sizes with support from a statistician to ensure methodological rigor [18]. Differences in study settings and fall outcomes (e.g., injurious falls) were considered during data synthesis.

### 2.6 Statistical Analysis

Meta‐analyses were performed when at least two studies reported comparable effect sizes for a specific exposure‐outcome association [19]. Given the methodological heterogeneity among studies, a random‐effects model was considered appropriate [20, 21]. Meta‐analyses were performed using IBM SPSS Statistics (Version 29, IBM Corp., Armonk, NY). Statistical heterogeneity was assessed using the I^2^ statistic. Studies with substantially different exposure measures or effect sizes were synthesized narratively. Sensitivity analyses were conducted to assess the robustness of pooled estimates [22]. As no category included 10 or more studies, formal assessment of publication bias was not conducted, in line with recommended methodological criteria [23].

### 2.7 Quality Assessment

Risk of bias was assessed using the National Institutes of Health (NIH) Quality Assessment Tool for Observational Cohort and Cross‐Sectional Studies [24]. This tool has been endorsed in recent systematic review methodology guidelines [25] and has been applied in prior reviews in this same field [7].

Quality ratings (good, fair, poor) were assigned individually based on the NIH assessment tool. Lack of adjustment for potential confounders was considered a key limitation and, in most cases, led to a rating of ‘poor quality’, given its impact on the internal validity and credibility of the results [24].

To minimize subjectivity, two reviewers (S.V. and G.M.) assessed study quality and cross‐checked their ratings to ensure consistency [26]. Discrepancies were resolved through discussion or consultation with a third reviewer (T.T.) [26].

## 3 Results

### 3.1 Study Selection

**Figure 1:**
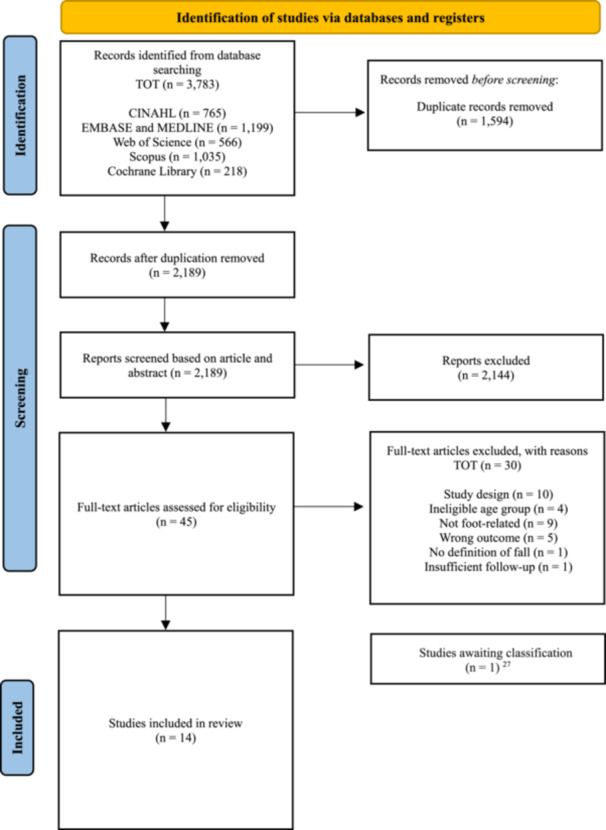
PRISMA flow diagram.

Database searches retrieved a total of 3,783 records. The study selection process is summarized in the PRISMA flow diagram [12] (Figure 1). Ultimately, 14 prospective observational studies met the eligibility criteria and were included. No additional studies were found through backward and forward reference searching.

### 3.2 Study Characteristics

The studies were published between 1988 and 2024 across seven countries (United States, Brazil, United Kingdom, Finland, Netherlands, Italy and Australia). Most recruited community‐dwelling older adults; one was conducted in residential care [27]. All studies included participants aged ≥ 60 years, with mean ages ranging from 67.6 to 80.1 years. The proportion of female participants ranged from 49% to 79.5% across studies with a female majority in 11 out of 14 studies. Sample sizes ranged from 71 to 3,617 (median = 303). The most frequent follow‐up duration was 12 months (8 out of 14 studies).

Fall definitions used in the included studies followed the criteria established by the World Health Organization (WHO) [15], the Kellogg International Work Group [28], the Prevention of Falls Network Europe (ProFaNE) [29], or Buchner et al. [30]. Fall outcomes were mainly ascertained through self‐reported fall calendars or phone contact; only three studies [9, 31, 32] used externally verified fall data. Additionally, two studies specifically assessed injurious falls rather than generic falls [32, 33].

Multiple foot and ankle domains were often assessed within the same study. The most frequently investigated foot‐related domains were foot deformities (5 studies) [9, 27, 33, 34, 35], foot sensory function (6 studies) [9, 27, 32, 34, 36, 37] and foot muscle strength (5 studies) [5, 9, 27, 38, 39] followed by foot pain (4 studies) [9, 27, 34, 40]. Four studies investigated ankle‐related domains: two assessed ankle muscle strength [5, 38] and two evaluated ankle range of motion [9, 27].

A full overview of study characteristics and assessment approaches is provided in Supporting Information S3.

### 3.3 Meta‐Analysis and Narrative Synthesis

An overview of the key results extracted from each included study, including effect estimates and exposure‐outcome associations, is provided in Supporting Information S4.

#### 3.3.1 Foot/Ankle Pain

Four studies [9, 27, 34, 40] investigated the association between foot pain and falls. Of these, three [9, 34, 40] were included in the meta‐analysis. One study [27] was excluded because foot pain was reported in a way that was not comparable to the others.

**Figure 2:**
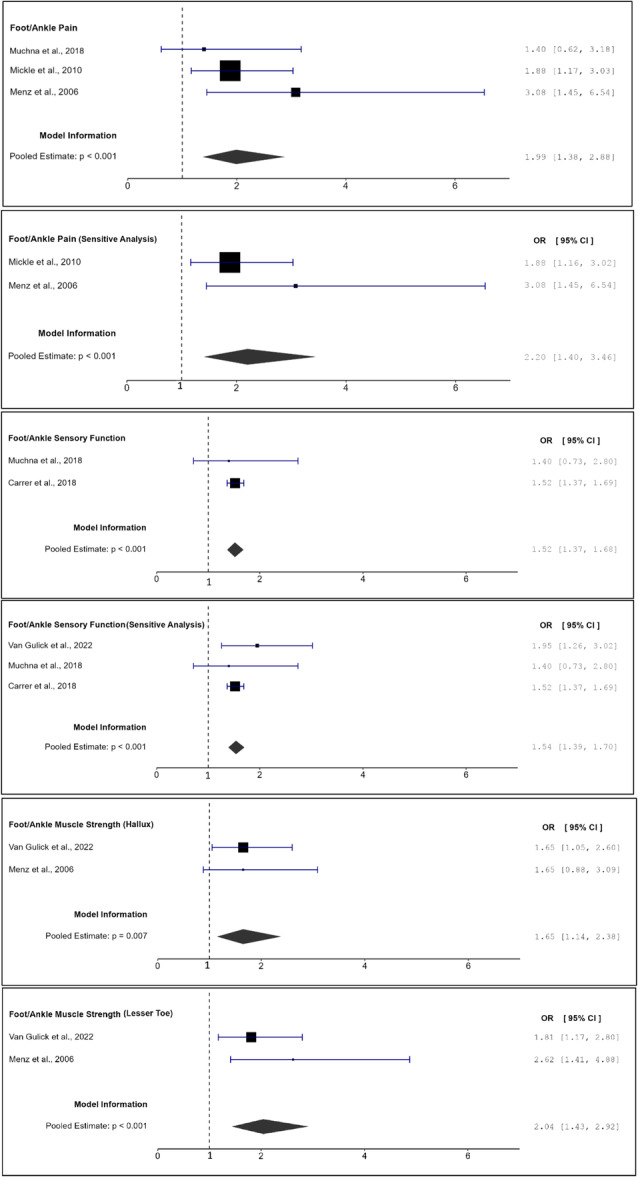
Forest plot of the pooled effect of specific foot and ankle problems and fall risk.

A random‐effects meta‐analysis of the three included studies showed a significant association between foot/ankle pain and fall risk (pooled OR = 1.99; 95% CI: 1.38–2.88; I^2^ = 2.6%) (Figure 2).

A sensitivity analysis excluding Muchna et al. [34], which differed in pain assessment and adjustment methods, produced a comparable effect (pooled OR = 2.20; 95% CI: 1.40–3.46; I^2^ = 15.1%), supporting the robustness of the findings (Figure 2).

#### 3.3.2 Foot/Ankle Sensory Function

Six studies [9, 27, 32, 34, 36, 37] assessed the association between sensory function and fall risk. A meta‐analysis was performed using data from two studies [34, 37]. For the study by Carrer et al. [37], only data from Model 1 (adjusted for age and sex), related to single falls and reduced foot sensation were extracted, to ensure comparability with the outcome and exposure definitions used in the other included study. A pooled analysis of these two studies showed a significant association between impaired sensory function and increased fall risk (pooled OR = 1.52; 95% CI: 1.37–1.68; I^2^ = 0%) (Figure 2).

A sensitivity analysis including the unadjusted estimate from Van Gulick et al. [27] yielded a similar pooled effect (pooled OR = 1.54; 95% CI: 1.39–1.70; I^2^ = 0%) (Figure 2).

Three studies [9, 32, 36] were excluded due to incompatible effect measures or outcome definitions. Menz et al. [9] reported standardized mean differences (SMDs), while Lipsitz et al. [36] reported relative risks (RR). Hicks et al. [32] used incidence rate ratios (IRR) and hazard ratios (HR) based on injurious falls coded according to the International Classification of Diseases (ICD). Nonetheless, all three studies reported findings consistent with an increased fall risk in individuals with sensory impairment, in line with the association observed in the meta‐analysis.

#### 3.3.3 Foot/Ankle Muscle Strength

Five studies [5, 9, 27, 38, 39] investigated the association between foot and ankle muscle strength and fall risk. A meta‐analysis was conducted on two studies [9, 27] assessing hallux plantarflexor strength using the paper grip test, showing a significant association with increased fall risk (pooled OR = 1.65; 95% CI: 1.14–2.38; I^2^ = 0%) (Figure 2).

A separate meta‐analysis on the same two studies [9, 27] evaluating lesser toe plantarflexor strength also showed a significant association with fall risk (pooled OR = 2.04; 95% CI: 1.43–2.92; I^2^ = 0%) (Figure 2).

Additionally, Mansi et al. [39] reported that increased hallux plantarflexor strength was significantly associated with lower fall risk, although this study was not included in the meta‐analysis due to methodological differences.

In contrast, two studies [5, 38] that assessed ankle dorsiflexor and plantarflexor muscle strength did not find significant associations with fall risk.

#### 3.3.4 Foot/Ankle Deformities

Five studies [9, 27, 33, 34, 35] assessed foot deformities in relation to fall risk, but heterogeneity in definitions, diagnostic criteria, and effect measures precluded meta‐analysis. Significant associations were observed in two studies: Mickle et al. [35] reported an increased risk of falls for severe hallux valgus (RR = 2.36; 95% CI: 1.03–5.45) and for lesser toe deformities (RR = 1.32; 95% CI: 1.04–1.69), while Menz et al. [9] found a modest association between hallux valgus severity and fall risk (SMD = 0.34; 95% CI: 0.04–0.64).

In contrast, Koski et al. [33] and Muchna et al. [34] did not identify significant associations between foot deformities and falls. Similarly, Van Gulick et al. [27] reported non‐significant findings for various grades of hallux valgus and lesser toe deformities, although confidence intervals were not provided.

#### 3.3.5 Foot/Ankle Range of Motion (ROM)

Two studies [9, 27] assessed range of motion in relation to falls. Menz et al. [9] reported a small but significant association for reduced ankle mobility (SMD = –0.32; 95% CI: –0.62 to –0.02; *p* = 0.041), but not for 1st metatarsophalangeal joint mobility. Van Gulick et al. [27] found no statistically significant association for ankle dorsiflexion.

#### 3.3.6 Foot/Ankle Posture

One study [9] assessed foot posture using three measures (Foot Posture Index, Arch Index and Navicular Height) and found no statistically significant associations with falls.

#### 3.3.7 Foot/Ankle Lesions

One study [9] examined foot lesions, reporting a non‐significant association between corns and falls.

#### 3.3.8 General Foot/Ankle Problems

In the included studies, general foot and ankle problems were defined as the presence of more than one foot or ankle problem, grouped and analyzed as a single exposure. Three studies [31, 34, 41] examined this domain. Only one study [31], conducted in a residential care setting, reported a significant association with falls (OR = 2.45; 95% CI: 1.35–4.44). The other two [34, 41] did not find statistically significant associations with falls.

### 3.4 Methodological Quality Assessment

Methodological quality was assessed using the NIH tool for observational cohort and cross‐sectional studies [24]. Four studies were rated “good” [5, 32, 36, 38] two “fair” [27, 37] and eight “poor” [9, 31, 33, 34, 35, 39, 40, 41]. Main concerns included insufficient adjustment for confounders and differences in how foot and ankle problems were assessed and defined.

**Figure 3:**
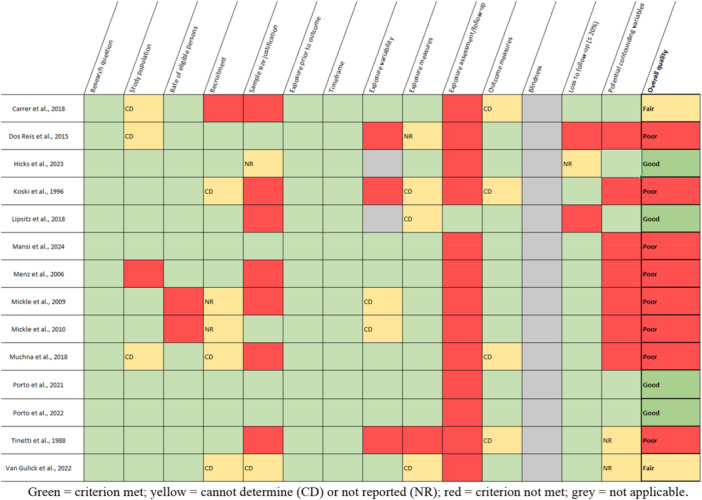
Methodological quality assessment of the included studies, using the NIH tool for observational and cross‐sectional studies [24]. Green = criterion met; yellow = cannot determine (CD) or not reported (NR); red = criterion not met; gray = not applicable.

**Figure 4:**
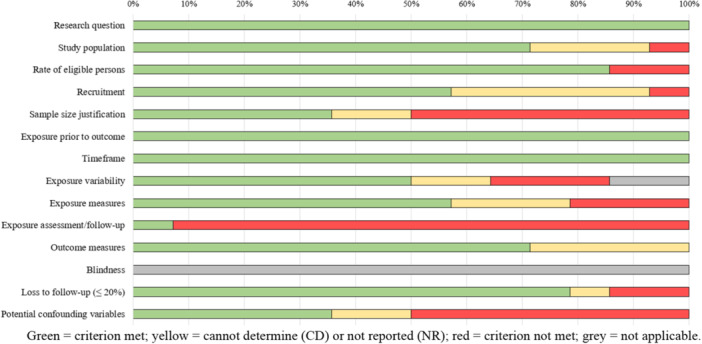
Percentage of risk of bias for included studies according to the NIH tool for observational and cross‐sectional studies [24]. Green = criterion met; yellow = cannot determine (CD) or not reported (NR); red = criterion not met; gray = not applicable.

The results of the quality assessment for individual studies are summarized in Figure 3, which provides a color‐coded overview of study quality. Figure 4 presents the percentage of studies meeting each individual quality criterion of the NIH tool.

## 4 Discussion

We synthesized evidence from 14 prospective observational studies examining foot and ankle problems as risk factors for falls among older adults. By including only prospective designs, this review strengthens causal inference [11] compared with earlier reviews and provides an updated picture of where evidence is robust and where it remains limited. While the scope of this review encompassed both the foot and the ankle, most of the evidence derived from meta‐analysis regarded foot‐related factors. We identified three domains (i.e., pain, impaired sensory function and reduced intrinsic foot muscle strength) showing the most consistent associations with falls. Other foot and ankle domains (i.e., deformities, posture, lesions, range of motion, general foot/ankle problems) showed less consistent or non‐significant associations with falls.

We found foot pain associated with almost twice the odds of falling, confirming it as a clinically meaningful contributor to fall risk. These findings align with Menz et al. [7], though our exclusive focus on prospective cohort studies strengthens the temporal inference. In the literature, pain is an established risk factor for falling in older adults interfering with cognition, executive function and psychological aspects, all factors that can increase the risk of falls in their own right [10, 42]. When located at the foot, pain may also impair balance and gait stability, offering a biomechanical explanation [43, 44, 45]. These findings reinforce foot pain as an independent contributor to fall risk in older adults.

Impaired foot sensory function demonstrated a consistent association with increased fall risk, confirmed by sensitivity analysis. Three additional studies [9, 32, 36] examining foot sensory function were not included in the meta‐analysis due to differences in effect measures, but all reported findings consistent with an increased fall risk. Hicks et al. [32] performed a standardized monofilament protocol in a large population‐based cohort and found that peripheral sensory loss was associated with increased incidence of injurious falls over 2.5 years of follow‐up (IRR = 1.64; 95% CI: 1.34–2.01), providing complementary evidence in support of the present findings. Additionally, Lipsitz et al. [36] conducted repeated exposure assessments during a 60‐month follow‐up and found that participants who developed sensory impairment over time had a significantly increased fall risk (adjusted RR = 1.57; 95% CI: 1.12–2.22). This supports the view that impaired sensory function may represent a dynamic and evolving risk factor. Therefore, because sensory function may develop or worsen over time, it may not be adequately captured by a single baseline assessment. Our findings represent a novel contribution, as this domain did not emerge in previous reviews [7]. The relevance of sensory loss as a fall‐related risk factor is already established in other clinical contexts. In diabetes care, for example, the clinical relevance of foot sensation in fall prevention is well‐recognized and annual monofilament testing is standard practice [46, 47, 48]. In contrast, there are no similar recommendations for older adults without diabetes, even though peripheral sensory deficits are also common in this population [49]. Based on the association observed in this review, routine and periodic assessment of foot sensation may be warranted. Monofilament testing, in particular, may represent a simple and low‐cost method to help identify individuals at increased risk of falling [32].

Finally, reduced foot muscle strength, particularly involving the hallux and lesser toes, was consistently associated with increased fall risk. Two studies [9, 27] using the paper grip test provided sufficiently comparable data to allow separate meta‐analysis for hallux and lesser‐toe strength, both indicating an increased likelihood of future falls. A third study [39] reported similar findings using a modified paper grip test with a dynamometer, but methodological differences did not allow its inclusion in the pooled analysis.

This association has important clinical implications, as foot muscle strength represents a modifiable risk factor. Strengthening exercises targeting intrinsic foot muscles have demonstrated improvements in balance and functional mobility in older adults [50], suggesting a plausible mechanism through which intervention might reduce fall risk. However, current systematic reviews do not show a significant reduction in fall incidence, and the overall quality of the available evidence remains low [51, 52]. Despite these gaps in intervention research, the prospective association demonstrated in this systematic review supports the clinical value of incorporating foot muscle strength assessment into comprehensive fall risk screening, enabling early identification of at‐risk individuals for targeted prevention strategies.

Other foot and ankle domains, including deformities, range of motion, lesions, posture, and general foot problems, were investigated in a limited number of studies and with considerable heterogeneity in definitions and methods, making it difficult to draw meaningful conclusions. Among these, hallux valgus and lesser toe deformities had previously shown significant associations with fall risk in the review by Menz et al. [7]. However, while current prospective evidence remains inconclusive, the biomechanical plausibility of these associations [50] suggests that further studies with clearer methodology and more comparable outcomes are warranted.

This review identified substantial heterogeneity across included studies regarding exposure definitions, diagnostic methods, and outcome ascertainment. For example, studies assessing sensory impairment used the Semmes‐Weinstein monofilament differently (single [27, 36] vs. multiple sites [34, 37, 48]), and some employed different tools like aesthesiometers [9]. Similar inconsistencies were observed for foot pain and muscle strength assessments, reflecting the broader lack of standardization in foot health assessment [53]. These variations may have introduced misclassification bias [54]. Moreover, all studies, except one [36], assessed exposures only at baseline without reassessment over time, which may have weakened the accuracy of exposure measurement for time‐varying conditions like pain, sensitivity or muscle strength. Furthermore, adjustment for confounding was generally inadequate: eight out of fourteen studies did not adjust for any variable, which significantly limits the interpretability of their findings [24]. Thus, the quality of the available evidence was generally low, which must be considered when interpreting the findings of this review. Finally, the small number of studies available for each meta‐analysis reduces the precision of pooled estimates and limits the reliability of the heterogeneity estimates (I^2^) [54].

Despite these limitations, this systematic review provides the most up‐to‐date prospective synthesis of foot and ankle risk factors for falls in older adults and identifies priorities for clinical practice and research. By including studies conducted in different settings, this review highlighted areas where research is limited: only one study was conducted in residential care facilities [31] and none in hospital settings. There is therefore a limited amount of research available in these populations, highlighting a knowledge gap in settings where the burden of falls is particularly high [55] and where further studies are urgently needed.

This review adds to the growing body of evidence supporting the role of foot problems as important risk factors for falls in older adults. In particular, foot pain, sensory impairment, and muscle weakness emerged as key contributors. These findings reinforce the need to integrate foot health assessment into fall prevention strategies. While current fall prevention guidelines [10] acknowledge the importance of foot assessment, they do not specify which aspects should be evaluated nor how they should be assessed. This review provides preliminary insights that may help inform a more targeted approach to clinical assessment and intervention on foot and ankle problems.

From a research perspective, future studies should prioritize using standardized, validated methods for the assessment of exposures and outcomes, include repeated measurements over time to better capture dynamic risk factors, and adjusting comprehensively for known confounders. Finally, developing and validating screening tools focused on foot‐related fall risk factors could help identify at‐risk individuals earlier and support targeted preventive intervention.

## Author Contributions

**Samuele Valeriani:** conceptualization, investigation, writing – original draft, methodology, writing – review and editing, project administration, validation. **Gianluca Melotto:** supervision, validation, methodology, writing – review and editing. **Thanaporn Tunprasert:** supervision, validation, methodology, conceptualization, writing – review and editing.

## Conflicts of Interest

The authors declare no conflicts of interest.

## Transparency Statement

The Lead author (Samuele Valeriani) and the Corresponding author (Thanaporn Tunprasert) affirm that this manuscript is an honest, accurate, and transparent account of the study being reported; that no important aspects of the study have been omitted; and that any discrepancies from the study as planned (and, if relevant, registered) have been explained.

## Supporting information


Supporting File

## Data Availability

The data that support the findings of this study are available in the Supporting information of this article.
