# Supplementary material for: “Foot and Ankle Risk Factors for Future Falls in Older Adults: A Systematic Review With Meta‐Analysis of Prospective Cohort Studies”
Source: Health Sci Rep. 2026 Aug 13;9(8):e73007. doi: 10.1002/hsr2.73007 (PMC13473745; doi:10.1002/hsr2.73007)
Supplement: Supplementary file 1 [file HSR2-9-e73007-s001.docx]

**S1. Deviations from the protocol**

| **Sections where occurred** | **Deviation** | **Justification** |
| --- | --- | --- |
| Quality Assessment Tool | Quality assessment tool changed from the Newcastle–Ottawa Scale (NOS) to the National Institute of Health (NIH) Quality Assessment Tool for Observational Cohort and Cross-Sectional Studies. | The NIH tool was selected because it was considered more appropriate for the aims of this review, as it allows a more detailed study-level assessment than the NOS. It covers a broader range of methodological aspects relevant to observational studies.  Furthermore, its use in prior reviews in the same field supports methodological consistency and facilitates comparison with existing evidence. |

**S2. Search strategy for the different databases**

*Search strategy for OVID (used to access MEDLINE and EMBASE) (01-06-2024)*

| **No.** | **Search terms/combinations** | **Results** |
| --- | --- | --- |
| #1 | (older OR elderly OR senior* OR aged OR ag?ing OR geriatr*).ab,kf,ti. | 3,992,116 |
| #2 | Exp Aged/ | 7,359,135 |
| #3 | #1 OR #2 | 9,799,740 |
| #4 | (problem* OR disorder* OR disease* OR condition* OR pain OR postur* OR characteristic* OR deformit* OR weak* OR instabilit* OR dysfunction* OR sensitivity OR type* OR flat OR pronat* OR supinat*).ab,kf,ti. | 31,948,444 |
| #5 | fall*.ab,kf,ti. | 599,153 |
| #6 | Exp accidental falls/ | 81,033 |
| #7 | #5 OR #6 | 625,504 |
| #8 | (foot OR feet OR ankle* OR toe* OR hallux OR hallucis OR heel*).ab,kf,ti. | 546,521 |
| #9 | Foot/ OR exp Foot Deformities/ OR exp Foot Diseases/ OR exp Toes/ | 236,282 |
| #10 | #8 OR #9 | 630,823 |
| #11 | ((epidemiologic adj (study OR studies)) OR (cohort adj (study OR studies)) OR cohort analy$ OR (follow up adj (study OR studies)) OR longitudinal OR prospective$ OR (observ$ adj3 (study OR studies))).ab,ti. | 7,740,157 |
| #12 | Epidemiologic Studies/ OR exp Cohort Studies/ | 4,038,545 |
| #13 | #11 OR #12 | 9,285,577 |
| #14 | #3 AND #4 AND #7 AND #10 AND #13 | 1,199 |

*Search strategy for SCOPUS (01/06/2024)*

| **No.** | **Search terms/combinations** | **Results** |
| --- | --- | --- |
| #1 | TITLE-ABS-KEY (older OR elderly OR senior* OR aged OR ag?ing OR geriatr*) | 7,212,269 |
| #2 | TITLE-ABS-KEY (problem* OR disorder* OR disease* OR condition* OR pain OR postur* OR characteristic* OR deformit* OR weak* OR instabilit* OR dysfunction* OR sensitivity OR type* OR flat OR pronat* OR supinat*) | 39,629,765 |
| #3 | TITLE-ABS-KEY (fall*) | 830,131 |
| #4 | TITLE-ABS-KEY (foot OR feet OR ankle* OR toe* OR hallux OR hallucis OR heel*) | 503,948 |
| #5 | TITLE-ABS-KEY (epidemiologic* OR cohort* OR "follow up" OR longitudinal OR prospective* OR observational) | 5,049,580 |
| #6 | #1 AND #2 AND #3 AND #4 AND #5 | 1,035 |

*Search strategy for EBSCOhost (used to access CINAHL) (01-06-2024)*

| **No.** | **Search terms/combinations** | **Results** |
| --- | --- | --- |
| #1 | TX (older OR elderly OR senior* OR aged OR ag?ing OR geriatr*) | 1,846,069 |
| #2 | (MH "Aged+") | 963,656 |
| #3 | #1 OR #2 | 1,846,100 |
| #4 | TX (problem* OR disorder* OR disease* OR condition* OR pain OR postur* OR characteristic* OR deformit* OR weak* OR instabilit* OR dysfunction* OR sensitivity OR type* OR flat OR pronat* OR supinat*) | 3,544,751 |
| #5 | TX fall* | 114,252 |
| #6 | (MH "Accidental Falls") | 26,851 |
| #7 | #5 OR #6 | 114,252 |
| #8 | TX (foot OR feet OR ankle* OR toe* OR hallux OR hallucis OR heel*) | 106,782 |
| #9 | (MH "Foot") OR (MH "Foot Diseases+") OR (MH "Foot Deformities+") OR (MH "Toes") OR (MH "Ankle") OR (MH "Heel") | 30,460 |
| #10 | #8 OR #9 | 107,987 |
| #11 | TX (epidemiologic* OR cohort* OR "follow up" OR longitudinal OR prospective* OR observational) | 1,110,466 |
| #12 | (MH "Epidemiological Research") OR (MH "Prospective Studies+") | 557,498 |
| #13 | #11 OR #12 | 1,130,623 |
| #14 | #3 AND #4 AND #7 AND #10 AND #13 | 765 |

*Search strategy for Web Of Science (WOS) (01/06/2024)*

| **No.** | **Search terms/combinations** | **Results** |
| --- | --- | --- |
| #1 | TS=(older OR elderly OR senior* OR aged OR ag$ing OR geriatr*) | 5,983,367 |
| #2 | TS=(problem* OR disorder* OR disease* OR condition* OR pain OR postur* OR characteristic* OR deformit* OR weak* OR instabilit* OR dysfunction* OR sensitivity OR type* OR flat OR pronat* OR supinat*) | 26,523,705 |
| #3 | TS=(fall*) | 539,278 |
| #4 | TS=(foot OR feet OR ankle* OR toe* OR hallux OR hallucis OR heel*) | 317,194 |
| #5 | TS=(epidemiologic* OR cohort* OR "follow up" OR longitudinal OR prospective* OR observational) | 3,601,841 |
| #6 | #1 AND #2 AND #3 AND #4 AND #5 | 566 |

*Search strategy for COCHRANE Library (01/06/2024)*

| **No.** | **Search terms/combinations** | **Results** |
| --- | --- | --- |
| #1 | (older OR elderly OR senior* OR aged OR ag?ing OR geriatr*):ti,ab,kw | 711,312 |
| #2 | MeSH descriptor: [Aged] explode all trees | 274,545 |
| #3 | #1 OR #2 | 711,312 |
| #4 | (problem* OR disorder* OR disease* OR condition* OR pain OR postur* OR characteristic* OR deformit* OR weak* OR instabilit* OR dysfunction* OR sensitivity OR type* OR flat OR pronat* OR supinat*):ti,ab,kw | 1,271,262 |
| #5 | (fall*):ti,ab,kw | 28,925 |
| #6 | MesH descriptor: [Accidental Falls] explode all trees | 2,160 |
| #7 | #5 OR #6 | 28,925 |
| #8 | (foot OR feet OR ankle* OR toe* OR hallux OR hallucis OR heel*):ti,ab,kw | 34,430 |
| #9 | MeSH descriptor: [Foot] this term only | 1,251 |
| #10 | MeSH descriptor: [Foot Deformities] explode all trees | 656 |
| #11 | MeSH descriptor: [Foot Diseases] explode all trees | 1,797 |
| #12 | MeSH descriptor: [Toes] explode all trees | 192 |
| #13 | #9 OR #10 OR #11 OR #12 | 3,712 |
| #14 | #8 OR #13 | 34,739 |
| #15 | (epidemiologic* OR cohort* OR "follow up" OR longitudinal OR prospective* OR observational):ti,ab,kw | 598,167 |
| #16 | MeSH descriptor: [Epidemiologic Studies] this term only | 129 |
| #17 | MeSH descriptor: [Cohort Studies] explode all trees | 210,984 |
| #18 | #16 OR #17 | 211,083 |
| #19 | #15 OR #18 | 606,011 |
| #20 | #3 AND #4 AND #7 AND #14 AND #19 | 218 |

**S3. Study Characteristics**

| Authors and Year | Country - Patient Setting | Sample Size (n) | Sex | Mean Age ± SD (range) | - Definition of Fall  - Fall Assessment Methods  - Follow-Up Duration | Foot/Ankle Problems Assessed (Specific Parameter) | Assessment Method | Assessment Details |
| --- | --- | --- | --- | --- | --- | --- | --- | --- |
| Carrer et al.^37^, 2018 | Italy – Community | 1885 | 1050 female (55.7%) | 75.0 ± 7.3 (≥ 65) | - WHO^15^  - Self-Reported (face-to-face interviews)  - 53 months | *Sensory Function* (Tactile Sensitivity) | Semmes-Weinstein 10g Monofilament | 3 sites tested: hallux, 1^st^ and 5^th^ metatarsals; failure to detect ≥1 site = peripheral neuropathy; unilateral and bilateral impairment classified separately |
| Dos Reis et al.^32^, 2015 | Brazil – Residential Care | 271 | 156 female (57.76%) | 79.1 ± 9.7 (60 to 111) | - Own definition*  - Staff Monitoring (Report books) - 6 months | *General Foot/Ankle Problems* | Classified according to NANDA-I nursing assessment |  |
| Hicks et al. ^48^, 2023 | United States of America - Community | 3617 | 2141 female (59.2%) | 79.4 ± 4.7 (NR but cohort restricted to older adults) | - International Classification of Diseased (ICD) ICD-9, ICD-10 and ICD-Clinical Modification E codes  - Validated administrative data  - 30 months (Median) | *Sensory Function* (Tactile Sensitivity) | Semmes-Weinstein 10g Monofilament | 3 sites tested per foot: hallux, 1^st^ and 5^th^ metatarsals, each tested three times; a site was classified as insensate if ≥2/3 responses were incorrect; failure to detect ≥1 site = peripheral neuropathy |
| Koski et al.^33^, 1996 | Finland - Community | 843 | 602 female (71.4%) | / (≥ 70) | - WHO^15^  - Self-Reported (Diary and 3-monthly phone calls) - 24 months | *Deformity* (Hallux valgus) | / |  |
|  |  |  |  |  |  | *Muscle Strength* (Tibialis anterior; Tibialis posterior) | / |  |
| Lipsitz et al.^36^, 2018 | United States of America - Community | 351 | 230 female (66%) | 78 ± 5 (≥ 65) | - Kellogg^29^ - Self-Reported (Monthly falls calendar) - 60 months | *Sensory Function* (Tactile sensitivity) | Abbreviated Semmes-Weinstein 10g Monofilament | Tested at single site (dorsum of hallux); supine position; eyes closed; 4 trials per monofilament; failure to detect = peripheral neuropathy |
| Mansi et al.^39^, 2024 | United Kingdom - Community | 71 | 35 female (49%) | 69 median (65 to 79) | - ProFaNE^30^ - Self-Reported (Monthly phone contact) - 12 months | *Muscle Strength* (Hallux flexor) | Enhanced Paper Grip Test (EPGT) | Force measured under hallux using paper card + dynamometer; with and without ankle support; 3 trials per foot |
| Menz et al.^9^, 2006 | Australia - Community | 175 | 120 female (68%) | 80.1 ± 6.4 (62 to 96) | - Kellogg^29^ - Self-Reported + Nurse Verification (Monthly fall calendar and post fall interview) - 12 months | *Pain* | Manchester Foot Pain and Disability Index (MFPDI) | Current foot pain ≥1 month + ≥1 item on MFPDI questionnaire |
|  |  |  |  |  |  | *Deformity* (Hallux valgus; Lesser digital deformity) | Manchester scale (for hallux valgus) Clinical observation (for lesser digital deformities) | Visual rating using standardized photos |
|  |  |  |  |  |  | *Lesions* (Corns; Calluses) | Clinical observation | Inspection for corns, calluses summed across both feet |
|  |  |  |  |  |  | *Sensory Function* (Tactile Sensitivity) | Aesthesiometer | Two-alternative forced-choice tactile sensitivity test at 1st metatarsophalangeal joint |
|  |  |  |  |  |  | *Muscle Strength* (Hallux and Lesser toes flexor) | Paper Grip Test | Test performed once per foot; failure = card pulled away easily despite resistance |
|  |  |  |  |  |  | *Posture* | Foot Posture Index Arch Index Navicular Height |  |
|  |  |  |  |  |  | *Range of Motion* (Ankle dorsiflexion) | Goniometer during Modified Lunge Test (weight bearing) Goniometer (non-weight bearing) |  |
| Mickle et al.^35^, 2009 | Australia - Community | 303 | 149 female (49%) | / (60 to 90) | - Kellogg^29^ - Self-Reported (Monthly fall calendar collected at the end of each month) - 12 months | *Deformity* (Hallux valgus) | Manchester scale | Visual rating using standardized photos |
| Mickle et al.^40^, 2010 | Australia - Community | 303 | 149 female (49%) | / (60 to 90) | - Kellogg^29^ - Self-Reported (Monthly fall calendar collected at the end of each month) - 12 months | *Pain* | Manchester Foot Pain and Disability Index (MFPDI) | Self-reported; MFPDI score ≥1 = foot pain |
| Muchna et al.^34^, 2018 | United States of America - Community | 117 | 93 female (79.5%) | 79.1 ± 8.5 (≥ 65) | - ProFaNE^30^ - Self-Reported (Weekly fall diary collected at the end of the follow-up period) - 6 months | *Pain* | 10cm VAS scale | Self-reported; highest foot score used; VAS >4 = moderate-to-severe foot pain |
|  |  |  |  |  |  | *Deformity* (Hallux valgus; lesser digital deformities; Flatfoot) | Clinical observation | Visual inspection |
|  |  |  |  |  |  | *Sensory Function* (Tactile and vibration sensitivity) | Semmes-Weinstein 10g Monofilament + Vibratip 25V | Touch (10g monofilament) + vibration (25V VibraTip) at metatarsal & heel; failure to detect either = peripheral neuropathy |
|  |  |  |  |  |  | *General Foot/Ankle Problems* | / | Defined as presence of at least one among: VAS >4, peripheral neuropathy, or deformity |
| Porto et al.^38^, 2021 | Brazil - Community | 101 | 78 female (77.2%) | 67.6 ± 5 (60 to 80) | - Buchner et al.^31^, 1997 - Self-Reported (Monthly phone contact) - 12 months | *Muscle Strength* (Ankle dorsiflexors and plantarflexors) | Isokinetic dynamometer (Biodex Systema 4 Pro, New York, USA) | Peak torque of ankle (dominant limb); 3× 5s isometric contractions |
| Porto et al.^5^, 2022 | Brazil - Community | 100 | 77 female (77%) | 67.6 ± 5 (≥ 60) | - Buchner et al.^31^, 1997 - Self-Reported (Monthly phone contact) - 12 months | *Muscle Strength* (Ankle dorsiflexors and plantarflexors) | Isokinetic dynamometer (Biodex Systema 4 Pro, New York, USA) | Rate of torque development and Torque steadiness of ankle (dominant limb); 3× 5s isometric + 2× 15s submax contractions |
| Tinetti et al.^41^, 1988 | United States of America - Community | 336 | female (55%) | 78.3 ± 5.1 (≥75) | - Kellogg^29^ - Self-Reported (Fall diary and bimonthly phone contact) - 12 months | *General Foot/Ankle Problems* | / | Defined as presence of at least one among: Moderate or severe bunions; Toe deformities; Ulcers; Deformed nails |
| Van Gulick et al.^28^, 2022 | Netherlands - Community | 407 | 246 female (60.4%) | 74.3 ± 5.8 (≥ 65) | - ProFaNE^30^ - Self-Report (Monthly fall calendar collected at the end of each month and monthly phone contact) - 12 months | *Pain* | Foot and Ankle Outcome Score (FAOS) - pain subscale | Self-reported; highest foot score used; score >2 = moderate-to-severe pain |
|  |  |  |  |  |  | *Deformity* (Hallux valgus; Lesser digital deformity) | Manchester scale | Visual rating using standardized photos |
|  |  |  |  |  |  | *Sensory Function* (Tactile Sensitivity) | Semmes-Weinstein 10g Monofilament | Tested at single site (1st MPJ); eyes closed; 3 trials per foot; failure to detect >2 times = peripheral neuropathy |
|  |  |  |  |  |  | *Muscle Strength* (Hallux and Lesser toes flexor) | Paper Grip Test | Test performed once per foot; failure = inability to grip standard card for 3 attempts; both feet tested |
|  |  |  |  |  |  | *Range of Motion* (Ankle dorsiflexion) | Goniometer during Modified Lunge Test (weight bearing) | Smallest value from both ankles used |

*Notes*:

* Defined by Dos Reis et al., 2015 as “an unintentional event that results in changing the position of the individual to a lower level in relation to their initial position”

/ = Information not reported by the authors

*Abbreviations*: MFPDI = Manchester Foot Pain and Disability Index; NANDA-I = North American Nursing Diagnosis Association International; PRoFaNE = Prevention of Falls Network Europe; VAS = Visual Analogue Scale; WHO = World Health Organization

**S4. Study Results**

| Authors and Year | Foot/Ankle Problems Assessed | Unadjusted Study Results | Adjusted For | Adjusted Study Results | Statistical significance |
| --- | --- | --- | --- | --- | --- |
| Carrer et al.^37^, 2018 | *Sensory Function* (Tactile Sensitivity) | / | Model 1: Age, Sex  Model 2: Age, Sex, Geriatric Depression Scale score, body mass index, Mini-Mental State Examination score, educational level, monthly income, living alone, diabetes, orthostatic hypotension, cardiovascular diseases, vision deﬁcit, chronic obstructive pulmonary disease, fracture, and lower limb osteoarthritis, Romberg test result, number of drugs taken per day | • Peripheral Neuropathy Model 1*  **OR = 1.52 (1.37** – **1.69)**  • Peripheral Neuropathy Model 2*  **OR = 1.48 (1.33** – **1.65)** | **REACHED**  **REACHED** |
| Dos Reis et al.^32^, 2015 | *General Foot/Ankle Problems* | **OR = 2.45 (1.35** – **4.44)** | / | / | **REACHED** |
| Hicks et al. ^48^, 2023 | *Sensory Function* (Tactile Sensitivity) | *INJURIOUS FALLS*  **IRR = 1.64 (1.34 – 2.01)** | Model 1: Age, Sex, Race  Model 2: Age, Sex, race, education, Body Mass Index, smoking status, drinking status, physical activity, hypertension, hypercholesterolemia, cardiovascular disease, peripheral artery disease, chronic kidney disease, cancer, diabetes, cognitive status, antidepressant use, sedative use, hypnotic use, antipsychotic use, diuretic use, antiepileptic use, anticholinergic use, history of prior falls | *INJURIOUS FALLS*  • Peripheral Neuropathy Model 1  **HR = 1.53 (1.24 – 1.90)**  *INJURIOUS FALLS*  • Peripheral Neuropathy Model 2  **HR = 1.36 (1.09 – 1.69)** | **REACHED**  **REACHED** |
| Koski et al.^33^, 1996 | *Deformity* (Hallux valgus) | *INJURIOUS FALLS* RR = 1.1 (0.97 – 1.33)   *MAJOR INJURIES* OR = 1.9 (0.99 – 3.45) | / | / | NOT REACHED |
|  | *Muscle Strength* (Tibialis anterior; Tibialis posterior) | *INJURIOUS FALLS*  • Tibialis anterior  RR = 1.2 (0.97 – 1.42)  • Tibialis posterior  RR = 1.2 (0.97 – 1.37) | / | / | NOT REACHED |
| Lipsitz et al.^36^, 2018 | *Sensory Function* (Tactile sensitivity) | • Progressing to impairment  **RR = 1.60 (1.16** – **2.22)**  • Consistently impaired  RR = 1.39 (0.89 – 2.18) | Age, Sex, Average weekly physical activity (PASE), baseline Berg balance score, diabetes, comorbidity count, SPPB score | • Progressing to impairment  **Adjusted RR = 1.57 (1.12** – **2.22)**  • Consistently impaired  Adjusted RR = 1.48 (0.89 – 2.45) | **REACHED**  NOT REACHED |
| Mansi et al.^39^, 2024 | *Muscle Strength* (Hallux flexor) | **MinEPGT** • Ankle held  OR = 0.77 (0.64 – 0.94)  • Ankle NOT held  OR = 0.67 (0.51 – 0.86)  **MaxEPGT** • Ankle held  OR = 0.73 (0.59 – 0.89)  • Ankle NOT held  OR = 0.85 (0.72 – 0.99)  **AvgEPGT** • Ankle held  OR = 0.71 (0.57 – 0.88)  • Ankle NOT held  OR = 0.70 (0.56 – 0.88)  **DiffEPGT** • Ankle held  OR = /  • Ankle NOT held  **OR = 16.37 (2.54 – 105.30)** | / | / | NOT REACHED  NOT REACHED  NOT REACHED  NOT REACHED  NOT REACHED  NOT REACHED  NOT REACHED  **REACHED** |
| Menz et al.^9^, 2006 | *Pain* | **OR† = 3.08 (1.45** – **6.53)** | / | / | **REACHED** |
|  | *Deformity* (Hallux valgus; Lesser toe deformity) | • Hallux Valgus Severity  **SMD‡ = 0.34 (0.04** – **0.64)**  • Number of Lesser Toe Deformities  SMD‡ = 0.26 (-0.04 – 0.57) | / | / | **REACHED**  NOT REACHED |
|  | *Lesions* (Corns) | OR† = 0.88 (0.30 – 2.53) | / | / | NOT REACHED |
|  | *Sensory Function* (Tactile Sensitivity) | **SMD‡ = 0.36 (0.06** – **0.67)** | / | / | **REACHED** |
|  | *Muscle Strength* (Hallux and Lesser toes flexor) | • Failed Paper Grip Test (Hallux)  OR† = 1.65 (0.88 – 3.09)  • Failed Paper Grip Test (Lesser Toes)  **OR† = 2.62 (1.41** – **4.88)** | / | / | NOT REACHED  **REACHED** |
|  | *Posture* | • Foot Posture Index  SMD‡ = 0.05 (-0.25 – 0.35)  • Arch Index  SMD‡ = 0.00 (-0.30 – 0.30)  • Navicular height/Foot length  SMD‡ = -0.29 (-0.59 – 0.01) | / | / | NOT REACHED  NOT REACHED  NOT REACHED |
|  | *Range of Motion* | • Ankle Flexibility  **SMD‡ = -0.32 (-0.62** – **-0.02**)  • First Metatarsophalangeal Joint Range of Motion  SMD‡ = -0.03 (-0.33 – 0.27) | / | / | **REACHED**  NOT REACHED |
| Mickle et al.^35^, 2009 | *Deformity* (Hallux valgus; Lesser toe deformity) | • Severe Hallux Valgus  **RR = 2.36 (1.03** – **5.45)**  • Lesser Toe Deformity  **RR = 1.32 (1.04** – **1.69)** | / | / | **REACHED**  **REACHED** |
| Mickle et al.^40^, 2010 | *Pain* | **OR† = 1.88 (1.16 – 3.02)** | / | / | **REACHED** |
| Muchna et al.^34^, 2018 | *Pain* | / | Age | OR = 1.40 (0.60 – 3.10) | NOT REACHED |
|  | *Foot Deformity* (Hallux valgus; lesser digital deformities; Flatfoot) | / | Age | • Various Foot Deformities  Adjusted OR = 1.30 (0.66 – 2.40) | NOT REACHED |
|  | *Sensory Function* (Tactile and vibration sensitivity) | / | Age | Adjusted OR = 1.40 (0.73 – 2.80) | NOT REACHED |
|  | *General Foot/Ankle Problems* | • Any Foot/Ankle Problem  OR = 1.40 (0.74 – 2.50) | Age | • ≥2 Foot/Ankle Problems  Adjusted OR = 1.50 (0.76 – 3.10) | NOT REACHED |
| Porto et al.^38^, 2021 | *Muscle Strength* (Ankle dorsiflexors and plantarflexors) | • Peak Torque Ankle Plantar Flexors  OR = 0.36 (0.10, 1.19)  • **P**eak Torque Ankle Dorsiflexors  OR = 1.24 (0.02, 58.66) | Age, Sex, Number of comorbidities, Number of medications, Height, Timed Up and Go test | • Peak Torque Ankle Plantar Flexors  OR = 0.67 (0.15 – 2.88)   • Peak Torque Ankle Dorsiflexors  OR = 12.81 (0.11 – 1,381.86) | NOT REACHED  NOT REACHED |
| Porto et al.^5^, 2022 | *Muscle Strength* (Ankle dorsiflexors and plantarflexors) | • Rate of Torque 30ms Development Ankle Plantar Flexors  OR = 0.46 (0.09 – 2.34)  • Rate of Torque 200ms Development Ankle Plantar Flexors  OR **=** 0.99 (0.13 – 7.17)   • Rate of Torque Development 30ms Ankle Dorsiflexors  OR = 1.28 (0.38 – 4.29)  • Rate of Torque Development 200ms Ankle Dorsiflexors  OR = 0.27 (0.03 – 2.04)  • Torque Steadiness 10% Ankle Plantar Flexors  OR = 0.67 (0.26 – 1.67)  • Torque Steadiness 50% Ankle Plantar Flexors  OR = 1.02 (0.52 – 1.99)   • Torque Steadiness 10% Ankle Dorsiflexors  OR = 1.11 (0.37 – 3.34)  • Torque Steadiness 50% Ankle Dorsiflexors  OR = 1.30 (0.74 – 2.28) | Age, Sex, Number of comorbidities, Number of medications, Height, Timed Up and Go test | • Rate of Torque 30ms Development Ankle Plantar Flexors  Adjusted OR = 0.82 (0.13 – 4.90)  • Rate of Torque 200ms Development Ankle Plantar Flexors  Adjusted OR **=** 0.67 (0.08 – 5.61)    • Rate of Torque Development 30ms Ankle Dorsiflexors  Adjusted OR = 1.35 (0.34 – 5.25)  • Rate of Torque Development 200ms Ankle Dorsiflexors  Adjusted OR = 0.47 (0.06 – 3.71)   • Torque Steadiness 10% Ankle Plantar Flexors  Adjusted OR = 0.52 (0.14 – 1.87)  • Torque Steadiness 50% Ankle Plantar Flexors  Adjusted OR = 1.06 (0.48 – 2.33)    • Torque Steadiness 10% Ankle Dorsiflexors  Adjusted OR = 0.87 (0.22 – 3.50)  • Torque Steadiness 50% Ankle Dorsiflexors  Adjusted OR = 1.17 (0.59 – 2.29) | NOT REACHED  NOT REACHED  NOT REACHED  NOT REACHED  NOT REACHED  NOT REACHED  NOT REACHED  NOT REACHED |
| Tinetti et al.^41^, 1988 | *Foot Problems* (Moderate or severe bunions; Toe deformities; Ulcers; Deformed nails) | RR = 1.4 (1.0 – 1.8) | / | Adjusted OR = 1.8 (1.0 – 3.1) | NOT REACHED |
| Van Gulick et al.^28^, 2022 | *Pain* | / | / | • Foot pain 1  Adjusted OR = 1.21 ( / ) p value = .486  • Foot pain 2  Adjusted OR = 0.95 ( / ) p value = .830 | NOT REACHED  NOT REACHED |
|  | *Foot Deformity* (Hallux valgus; Lesser digital deformity) | / | / | • Hallux Valgus 1  Adjusted OR = 0.97 ( / ) p value = .916  • Hallux Valgus 2  Adjusted OR = 1.12 ( / ) p value = .727  • Hallux Valgus 3  Adjusted OR = 1.13 ( / ) p value = .781  • Lesser Toe Deformity  Adjusted OR = 1.03 ( / ) p value = .312 | NOT REACHED  NOT REACHED  NOT REACHED  NOT REACHED |
|  | *Sensory Function* (Tactile Sensitivity) | **OR† = 1.95 (1.26 - 3.02)** | / | **Adjusted** **OR = 1.77 ( / ) p value = .014** | **REACHED** |
|  | *Muscle Strength* (Hallux and Lesser toes flexor) | • Failed Paper Grip Test (Hallux)  OR† = 1.65 (1.05 – 2.60)  • Failed Paper Grip Test (Lesser Toes)  OR† = 1.81 (1.17 – 2.79) | / | • Failed Paper Grip Test (Hallux)  OR = 1.23 ( / ) p value = .458  • Failed Paper Grip Test (Lesser Toes**)**  OR = 1.45 ( / ) p value = .156 | NOT REACHED  NOT REACHED |
|  | *Range of Motion* (Ankle dorsiflexion) | / | / | Ankle-dorsiflexion ROM  OR = 0.98 ( / ) p value = .206 | NOT REACHED |

*Notes*:

* Based on the subgroup with single fall and impaired tactile sensitivity using the Semmes-Weinstein Monofilament Examination (SWME+ group).

† OR and 95% CI calculated by the review author from raw data using a 2×2 contingency table.

‡ SMD and 95% CI calculated by the review author from raw data using pooled standard deviation.

/ Information not reported by the authors

*Abbreviations*: OR = Odds Ratio; RR = Risk Ratio/Relative Risk; IRR = Incidence Rate Ratio; HR = Hazard Ratio; SMD = Standardized Mean Difference; ROM = Range of Motion; EPGT = Enhanced Paper Grip Test; MinEPGT = weakest limb EPGT force; MaxEPGT = strongest limb EPGT force; AvgEPGT = average between limbs EPGT force; DiffEPGT = normalised difference in strength between limbs
